# High incidence of thromboembolism in patients with chronic GVHD: association with severity of GVHD and donor-recipient ABO blood group

**DOI:** 10.1038/s41408-021-00488-2

**Published:** 2021-05-18

**Authors:** Najla El Jurdi, Heba Elhusseini, Joan Beckman, Todd E. DeFor, Grigori Okoev, John Rogosheske, Aleksandr Lazaryan, Kristen Weiler, Veronika Bachanova, Brian C. Betts, Bruce R. Blazar, Claudio G. Brunstein, Fiona He, Shernan G. Holtan, Murali Janakiram, Radhika Gangaraju, Joseph Maakaron, Margaret L. MacMillan, Armin Rashidi, Erica D. Warlick, Smita Bhatia, Gregory Vercellotti, Daniel J. Weisdorf, Mukta Arora

**Affiliations:** 1 Blood and Marrow Transplant Program, Department of Medicine, University of Minnesota, Minneapolis, MN USA; 2 Division of Hematology, Oncology, and Transplantation, Department of Medicine, University of Minnesota, Minneapolis, MN USA; 3 Biostatistics and Informatics, Clinical and Translational Science Institute, University of Minnesota, Minneapolis, MN USA; 4 Department of Pediatrics, University of Alabama, Tuscaloosa, AL USA

**Keywords:** Medical research, Cancer

## Abstract

Chronic graft-versus-host disease (cGVHD) after allogeneic hematopoietic cell transplantation (HCT) is associated with systemic inflammation and endothelial dysfunction, increasing risk for thromboembolic events (TEE). In 145 adult recipients who developed cGVHD after a matched sibling or umbilical cord blood donor HCT from 2010 to 2018, 32(22%) developed at least 1 TEE event, and 14(10%) developed 2 TEE events. The 5-year cumulative incidence of TEE was 22% (95% CI, 15–29%) with a median time from cGVHD to TEE of 234 days (range, 12–2050). Median time to the development of LE DVT or PE was 107 (range, 12–1925) compared to 450 days (range, 158–1300) for UE DVT. Cumulative incidence of TEE was 9% (95% CI, 0–20%), 17% (95% CI, 9–25%), and 38% (95% CI, 22–55%) in those with mild, moderate, and severe GVHD, respectively. Higher risk for TEE was associated with cGVHD severity (hazard ratio [HR] 4.9, [95% CI, 1.1–22.0]; *p* = 0.03), non-O-donor to recipient ABO match compared to O-donor to O-recipient match (HR 2.7, [95% CI, 1.0–7.5]; *p* = 0.053), and personal history of coronary artery disease (HR 2.4, [95% CI, 1.1–5.3]; *p* = 0.03). TEE was not associated with 2-year non-relapse mortality or 5-year overall survival.

## Key points

- Patients with chronic GVHD after allogeneic hematopoietic cell transplantation are at high risk for thromboembolic events occurring years after diagnosis.
- More severe chronic GVHD, non-O donor-recipient ABO compared to O-O match and personal history of coronary artery disease are associated with higher risk of thromboembolic events.

## Introduction

Chronic graft-versus-host disease (cGVHD) occurs in up to half of allogeneic HCT recipients, limits the success of allogeneic hematopoietic cell transplantation (HCT), and remains the leading cause of non-relapse mortality (NRM) and morbidity among survivors. cGVHD is a multisystem syndrome involving dysregulated immunity, tissue inflammation and injury, with endothelial dysfunction often resembling processes seen in autoimmune diseases and possibly leading to permanent organ damage^1–4^.

Venous and arterial thromboembolism is pathologic formation of thrombi in organs, often associated with inflammation. Individuals with other chronic autoimmune disorders are known to be at risk for TEE^5^. Endothelial dysfunction and decreased thrombomodulin- dependent generation of activated protein C have been implicated in GVHD pathogenesis, partially contributing to a procoagulant state^6–9^. Limited studies have reported a wide range of thromboembolism incidence among allogeneic HCT recipients^10–13^, with higher risk observed in patients developing GVHD^12,13^. Here, we aim to assess the incidence and risk factors for thromboembolic events (TEE), including venous thromboembolism (VTE) and pulmonary embolism (PE), among patients with known cGVHD and examine the impact of TEE on clinical outcomes after cGVHD.

## Methods

### Study design and inclusion criteria

The objective of this retrospective single-institution cohort study was to assess the incidence, risk factors, and clinical outcomes of patients with cGVHD who developed TEE. The study population included 145 consecutive adults who received their first allogeneic HCT and who developed cGVHD after a matched sibling (MSD) or umbilical cord blood (UCB) donor allogeneic HCT from 2010 to 2018 at the University of Minnesota. Bone marrow (BM), peripheral blood stem cell (PBSC), and UCB graft sources were included. Recipients received myeloablative (MAC) or reduced intensity conditioning (RIC) regimens. All GVHD prophylaxis strategies were included. All patients signed a written informed consent allowing the use of their medical data in clinical research analysis. This study was reviewed and approved by the University of Minnesota Institutional Review Board.

### Definitions

Thromboembolic events (TEE), included venous thromboembolism (VTE) and pulmonary embolism (PE), and were defined as any new event, confirmed by imaging and requiring systemic therapy at any time after cGVHD diagnosis. We categorized VTE sites as upper extremity (UE) or lower extremity (LE) proximal or distal deep vein thrombosis (DVT). UE DVT included line associated events, central or peripherally inserted central catheter, and grouped separately for analysis.

We followed the 2014 NIH Consensus Criteria for diagnosis, determining organ involvement and overall severity at diagnosis of cGVHD^14^. cGVHD at onset was categorized as de-novo if cGVHD developed without prior acute GVHD (aGVHD), quiescent if cGVHD developed after resolution of prior aGVHD, and progressive if cGvHD developed without resolution of prior aGVHD.

Non-relapse mortality (NRM) was defined as death in the absence of disease relapse or progression, accounting for relapse as a competing risk. Overall survival (OS) was defined as time from transplantation to death from any cause.

### Patient and transplant characteristics

The clinical factors examined for possible associations with TEE included: gender, age (<50 or ≥50), BMI (<30 or ≥30), donor type (MSD or UCB), conditioning intensity (MAC vs RIC), GVHD prophylaxis (cyclosporine [CsA] or tacrolimus [Tac] with methotrexate [MTX], CsA or Tac or sirolimus [Siro] with mycophenolate mofetil [MMF]), disease risk index for malignant disorders (DRI: low risk, intermediate risk, high/very risk, non-malignant was not included)^15^, HCT comorbidity index^16^ (HCT-CI: low risk, intermediate risk, high risk), type of cGVHD at onset (de-novo, quiescent or progressive), severity of cGVHD at onset (mild, moderate or severe), platelets at cGVHD diagnosis (<50,000, 50,000–100,000, >100,000), donor-recipient ABO match (O to O and non-O match groups for simplification given inclusion of double UCB), and cGVHD organ involvement (skin, eyes, mouth, joints, lung, gastrointestinal, genitourinary, liver). We additionally examined the effect of traditional TEE risk factors including smoking history, diabetes mellitus (DM), hyperlipidemia (HLD), hypertension (HTN), cerebrovascular accident (CVA), congestive heart failure (CHF), coronary artery disease (CAD), family history of TEE, and personal history of TEE prior to cGVHD diagnosis.

### Statistical analysis

We assessed the cumulative incidence of TEE after cGVHD treating non-TEE mortality as a competing risk^17^. Multivariate regression was used to evaluate the independent association of factors with the incidence of TEE^18^ using predetermined risk factors in our regression model including gender (male vs. female), age (<50 vs. ≥50), severity of cGVHD at onset (mild vs. moderate vs. severe), ABO blood group match (O to O vs. non-O match), history of CAD, and history of TEE prior to cGVHD. BMI and type of cGVHD (de-novo vs. quiescent vs. progressive) violated the proportional hazards assumption and were excluded from the regression model. Due to collinearity between organ involvement and cGVHD severity, only severity was included in the model. A separate model included cGVHD organ involvement at onset, mucocutanous (skin, oral and/or eye) vs. visceral. Given recurrent TEE episodes among some patients, the Prentice, Williams and Peterson model (PWP) for recurrent events was used to evaluate the independent association between the predetermined risk factors and TEE^19^. Cox and Fine and Gray regression models were used to evaluate the independent association of time-dependent VTE on OS and NRM, respectively, using propensity scoring to control for confounding^18,20^. Given the small number of events after censoring (30 for OS and 20 for NRM), analysis of the independent association of VTE on NRM and OS used a propensity score to control for confounding^21^.

All reported p-values were 2-sided. All analyses were performed using SAS 9.4 (SAS Institute, Inc., Cary, NC) and R version 3.6.2. Outcomes and covariates in regression models were all clinically pre-specified.

## Results

### Patient, treatment and cGVHD characteristics

Patient, disease, and transplant characteristics are shown in Table 1. A total of 145 patient who developed cGVHD were studied. Median age at time of cGVHD diagnosis was 52 years (range, 19–74). 104 (72%) patients received MSD and 41 (28%) single or double UCB allogeneic HCT. cGVHD was mild in severity in 24 patients (16%), moderate in 82 (57%), and severe in 39 (27%). Type of cGVHD was de-novo in 55 patients (38%), quiescent in 55 (38%), and progressive in 35 (24%). Patients developing TEE were more likely to have a history of CAD (44% vs. 19%) and prior history of TEE before cGVHD diagnosis (32% vs. 17%).

**Table 1:** Patient, disease and transplant characteristics.

	All patients frequency (%)
*N*	145
*Gender:* Male	95 (66%)
Age	
Median(range), (IQR)	52 (19–74), (41–62)
BMI	
Median(range), (IQR)	28.0 (19.2–50.2), (24.9–32.1)
Donor Type	
Matched Sibling	104 (72%)
Single UCB	9 (6%)
Double UCB	32 (22%)
Conditioning	
MAC	63 (43%)
RIC	82 (57%)
GVHD Prophylaxis	
CsA or TAC/MTX	51 (35%)
CsA or TAC/MMF	86 (59%)
Siro/MMF	8 (6%)
Diagnosis	
Acute Leukemia	86 (60%)
CML/CLL	6 (4%)
Lymphoma	25 (17%)
Other	28 (19%)
Karnofsky at HCT	
<90	20 (14%)
≥90	125 (86%)
HCT-CI	
Low Risk: 0	60 (41%)
Intermediate Risk: 1-2	42 (29%)
High Risk: 3+	43 (30%)
ABO Recipient	
A	62 (43%)
B	13 (9%)
AB	3 (2%)
O	67 (46%)
ABO Donor	
A, B or AB	75 (52%)
O	70 (48%)
Year of cGVHD	
2010–2013	76 (52%)
2014–2018	69 (48%)
Days from HCT to cGVHD	
Median(range), (IQR)	220 (88–1111), (168–309)
Karnofsky at cGVHD: <90	38 (26%)
cGVHD Type at Onset	
De-novo	55 (38%)
Quiescent	55 (38%)
Progressive	35 (24%)
cGVHD Global Severity at Onset	
Mild	24 (16%)
Moderate	82 (57%)
Severe	39 (27%)
Prior Acute GVHD Grade	
No acute GVHD	53 (37%)
Grade I–II	50 (34%)
Grade III–IV	42 (29%)
Number of Organs involved at cGVHD Onset	
1 or 2	75 (52%)
≥3	70 (48%)
*WBC at cGVHD Diagnosis*	
Median(range), (IQR) x10^9^/L	5.9 (1.2–17.2), (4.3–7.9)
Platelets at cGVHD Diagnosis	
Median(range), (IQR)	138 (3–470), (99–183)
<100,000 ×10^9^/L	38 (26%)
≥100,000 ×10^9^/L	107 (74%)
cGVHD Organ Involvement	
Skin	60 (40%)
Eyes	70 (48%)
Mouth	107 (74%)
Liver	42 (29%)
Gastrointestinal	59 (41%)
Genitourinary	7 (5%)
Lung	5 (4%)
Joints	15 (10%)
cGVHD Treatment	
Systemic	39 (27%)
Topical	12 (8%)
Both	94 (65%)

*BMI* body mass index, *TEE* thromboembolic events, *cGVHD* chronic graft-versus-host disease, *UCB* umbilical cord blood, *MAC* myeloablative, *RIC* reduced intensity, *CsA* cyclosporine, *Tac* tarolimus, *Siro* sirolimus, *MMF* mycophenolate mofetil, *MTX* methotrexate, *CML* chronic myeloid leukemia, *CLL* Chronic lymphocytic leukemia, *HCT* hematopoietic cell transplantation.

### TEE: incidence, subtype and timing after cGVHD

TEE characteristics are shown in Table 2. Of the 145 patients with cGVHD, 32 (22%) developed at least 1 TEE event, and 14 (10%) developed 2 TEE events. No patients developed more than 2 TEE events. For the first TEE event, 6 patients developed a PE (19%), 26 developed DVT (81%), and 1 patient developed a thrombus in the inferior vena cava. Location of DVT was LE in 17 patients and UE in 8 patients, with 5 of these 8 UE DVTs catheter-related. For the second TEE events, 2 patients developed a PE (14%), and 12 had a DVT (86%; *n* = 5 LE, *n* = 7 UE, and *n* = 4 of these 7 were catheter-related UE DVT). The cumulative incidence of TEE through 5 years post cGVHD diagnosis was estimated at 22% (95% CI, 15–29%) with a median time from cGVHD to TEE of 234 days (range, 12–2050; interquartile range [IQR] 85–599). Median time to the development of LE DVT or PE was 107 days (range, 12–1925), and median time to development of UE DVT was 450 days (range, 158–1300).

**Table 2:** Thromboembolism characteristics.

	1^st^ TEE Frequency (%)	2^nd^ TEE Frequency (%)
*N*	32	14
Median Days to Diagnosis of TEE from cGVHD (Range), (IQR)	233.5 (12–2050), (84.5–599)	715.5 (19–2137), (459–1139)
cGVHD Disease Status at TEE Event		
Inactive	5 (16%)	4 (29%)
Active	27 (84%)	10 (71%)
Type and Location of TEE		
PE	6 (19%)	2 (14%)
DVT	26 (81%)	12 (86%)
LE Left	9	3
LE Right	7	2
LE Bilateral	1	0
UE Left	4	2
UE Right	4	5
IVC	1	0
Occlusive DVT	16 (61%)	4 (33%)
UE Catheter Related DVT	5 (19%)	4 (33%)
Lab values at TEE Diagnosis		
Median (range), (IQR)		
WBC (x10^9^/L)	7.1 (0.6–21.2), (4.3–11.2)	6.7 (3.3–11.1), (5.2–9.3)
Absolute neutrophil count (x10^9^/L)	4.3 (0.6–14.6), (3.5–7.6)	4.3 (1.4–9.2), (3.5–7.2)
Absolute lymphocyte count (x10^9^/L)	1.0 (0–5.2), (0.5–1.8)	1.3 (0.3–9.5), (0.9–1.6)
Absolute eosinophil count (x10^9^/L)	0 (0–5.4), (0–0.1)	0.1 (0–0.7), (0–0.3)
Hemoglobin mg/dl	11.2 (7.4–15.3), (9.9–13.0)	12.0 (8.7–16.1), (10.5–14.4)
Platelet (x10^9^/L)	112 (8–346), (57–174)	136 (38–326), (61–184)
Lactate dehydrogenase U/L	437 (98–1122), (311–806)	384 (311–457)
Albumin	3.2 (1.5–4.2), (2.7–3.5)	3.1 (1.6–3.6), (2.5–3.4)
Immunosuppression Medications at and 60 days prior to TEE Number (proportion)		
Corticosteroids	28 (88%)	10 (71%)
Prednisone Dose equivalent mg/kg/day (averaged over 60 days prior to TEE); Median (range), (IQR)	0.3 (0.1–1.64), (0.2–0.5)	0.2 (0.04–1.6), (0.07–0.31)
Budesonide	3 (9%)	2 (14%)
Sirolimus	16 (50%)	4 (29%)
MMF	1 (3%)	0
Rituximab	2 (6%)	0
CNI	5 (16%)	2 (14%)
TNF-inhibitor	1 (3%)	1 (7%)
ECP	2 (6%)	1 (7%)
IVIG	11 (34%)	3 (21%)
Immunomodulatory Drugs	1 (3%)	0
Erythropoietin/Thrombopoietin	2 (6%)	0
Ibrutinib or ruxolitinib	2 (6%)	3 (21%)
Antiplatelet therapy (Aspirin)	3 (9%)	3 (21%)

*Duration expressed in median (range), (interquartile range).

*cGVHD* chronic graft-versus-host disease, TEE thromboembolic events, *PE* pulmonary embolism, *DVT* deep vein thrombosis, *LE* lower extremity, *UE* upper extremity, *IVC* inferior vena cava, *MMF* mycophenolate mofetil, *CNI* calcineurin inhibitor, *TNF* tumor necrosis factor, *ECP* extracorporeal photopheresis, *IVIG* intravenous immunoglobulin.

**Fig. 1:**
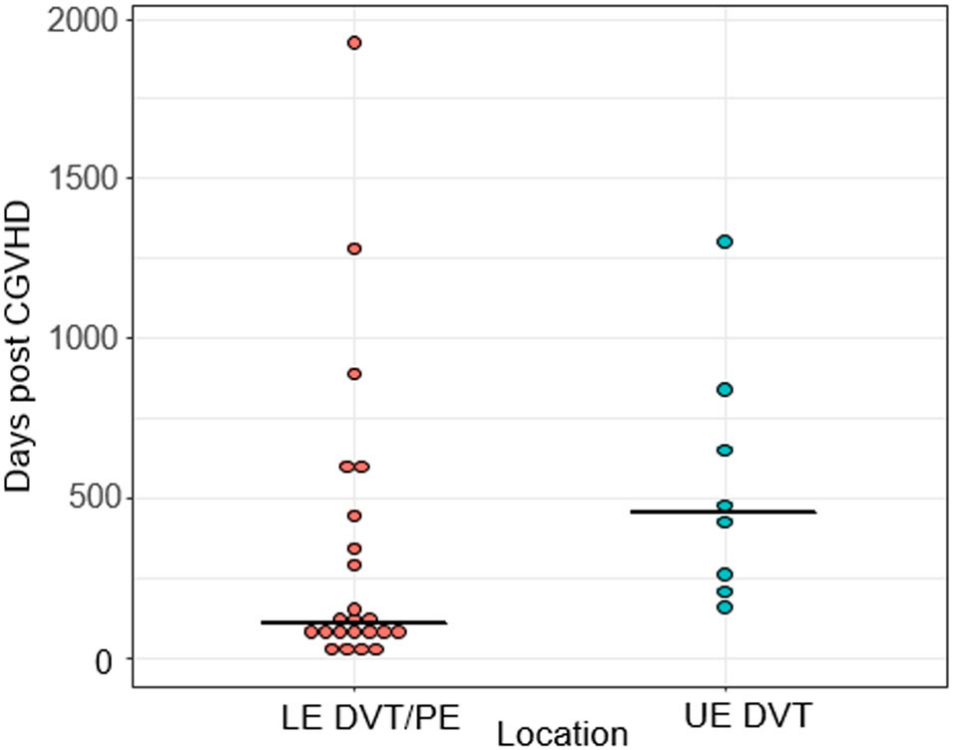
Dot plot of thromboembolic events. Each dot represents a unique event. Lower extremity (LE) deep vein thrombosis (DVT) events are displayed with pulmonary embolism (PE) events on the left. Upper extremity (UE) DVT events are displayed on the right.

Most patients were on corticosteroids at the time of first (*n* = 28, 88%) and second (*n* = 10, 71%) TEE, with a median prednisone dose equivalent to 0.3 and 0.2 mg/kg/day, respectively, averaged over 60 days prior to the TEE event. Sirolimus was the second most commonly used immunosuppression therapy (50 and 29% at first and second TEE, respectively). IVIG (intravenous immunoglobulin) was administered within 60 days of TEE in 11 (34%) and 3 (21%) patients at the time of first and second TEE, respectively. At the time of TEE, 3 patients were receiving aspirin. 4 (13%) and 8 (57%) patients developed first and second TEE, respectively, on prophylactic anticoagulation (due to history of prior TEE). Enoxaparin was the most commonly used anticoagulation therapy. Figure 1 is a dot plot of TEE by anatomic location and time post HCT.

### Risk factors for the development of TEE

Cumulative 5-year incidence of TEE was similar per gender (24% in males vs. 18% in females), age (23% vs. 21% with age <50 or ≥50), BMI (22% vs. 21% with BMI < 30 vs. ≥30), donor source (23% MSD vs. 20% UCB donor), conditioning (21% vs. 22% in MAC vs. RIC), GVHD prophylaxis (22% for CsA or Tac/MTX vs. CsA or Tac or Siro/MMF), DRI (19% in low and intermediate risk vs. 50% in high/very high risk), HCT-comorbidity index (22% in low and intermediate vs. 21% in high HCT-CI), or platelets at cGVHD diagnosis (31% vs. 23% vs. 21% for counts of <50,000 vs. 50,000–100,000 vs. >100,000). TEE incidence was 25% in patients with de novo cGVHD (95% CI, 13–37%) compared to 20 and 17% in those with quiescent (95% CI, 10–31%) or progressive type (95% CI, 5–30%), respectively. Cumulative incidence was 9% (95% CI, 0–20%), 17% (95% CI, 9–25%), and 38% (95% CI, 22–55%) in those with mild, moderate, and severe GVHD, respectively. When we examined the incidence per organ involvement, patients with lung cGVHD had a higher 5-year cumulative incidence TEE (60%, 95% CI [27–93%] vs. 20%, CI [13–27]). Donor-recipient ABO blood group O-O had lower 5-year cumulative incidence of TEE (11%; 95% CI, 1–22) compared to all other ABO matches combined (26%; 95% CI, 17–35).

**Fig. 2:**
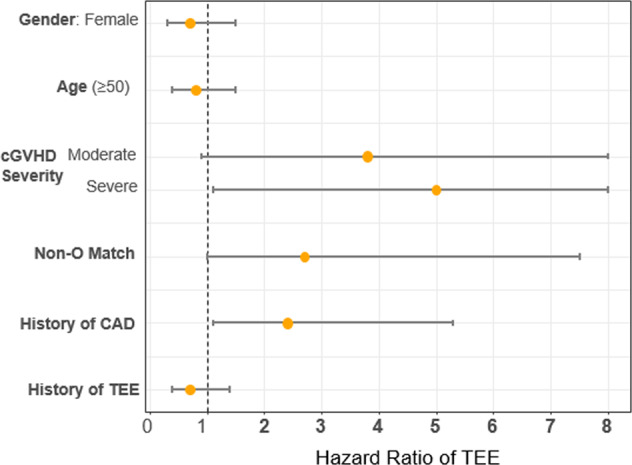
Multivariate regression of risk factors for thromboembolic event development among all patients with cGVHD. cGVHD = chronic graft-versus-host disease; CAD = coronary artery disease. Non-O match = includes all donor-recipient ABO match other than O to O.

In multivariate regression, we examined the independent impact of clinical factors on the development of TEE using the stated predetermined risk factors (Fig. 2). Higher risk for TEE was associated with cGVHD severity (hazard ratio [HR] 4.9, [95% CI, 1.1–22.0]; *p* = 0.03), non-O-donor to recipient ABO match compared to O-donor to O-recipient match (HR 2.7, [95% CI, 1.0–7.5]; *p* = 0.053), and personal history of coronary artery disease (HR 2.4, [95% CI, 1.1–5.3]; *p* = 0.03). Organ involvement at cGVHD onset (mucocutanous vs. visceral) was not associated with risk of subsequent TEE.

### TEE effect on non-relapse mortality and survival

TEE was not associated with 2-year NRM (HR 1.2, [95% CI; 0.4–3.6]) or 5-year OS (HR 1.4, [95% CI, 0.7–3.0]). The 5-year NRM and OS were not significantly different per TEE location. Disease relapse was the predominant cause of death in 55% of patients developing TEE compared to 34% in those without TEE. Relapses occurred in 5 patients before TEE diagnosis and in 7 patients after TEE with median time to relapse 302 days (range, 128–1231) after TEE. cGVHD was the primary cause of death in 11% of those developing TEE compared to 20% in those without TEE.

## Discussion

cGVHD is a complication after allogeneic HCT that is associated with high risk for developing thromboembolic complications. The risk of developing LE DVT or PE occur earlier than UE DVT; however, both can occur years after cGVHD diagnosis and require high levels of clinical attention. Our study showed that cGVHD severity, non-O donor-recipient ABO group match, and personal history of CAD are associated with higher risk of TEE development after cGVHD.

cGVHD is a cytokine-driven and immune-mediated complication resulting in systemic inflammation and endothelial dysfunction. Endothelial structure and function are central to orchestrating inflammatory and thrombotic responses. Cytotoxic T-lymphocytes and inflammatory cytokines contribute to endothelial injury after allogeneic HCT, which in turn can lead to impaired tissue perfusion and fibrosis^3,22,23^. Evidence of endothelial activation and damage can be found early post transplantation and is central to GVHD and other endothelial-driven complications after allogeneic HCT^2,24–26^. Additionally, further understanding of altered primary and secondary hemostasis after cGVHD will be critical to balance the increased risk of bleeding with the benefit of thromboprophylaxis in this population^10,12^.

There is a well-known association between ABO blood group and risk of thrombosis; particularly, those with a non-O blood group are at higher risk of arterial and venous thrombosis^27–29^. This increased risk is partially due to qualitative and quantitative differences in the glycoprotein von Willebrand factor (vWF), including 25% higher plasma levels of vWF in non-O blood group individuals^30,31^. VWF is not required for retention of red blood cells in clots^32^. However, ABO blood group contributes to VWF proteolysis and clearance and may contribute to VWF interactions with platelet glycoprotein Ib-IX-V and glycoprotein IIb-IIa complexes on platelet surfaces^33–35^. Other potential mechanisms for increased TEE post-transplant may include hemolysis, increased transfusions secondary to delayed RBC engraftment, and changes in endothelial cells^36^. In our cohort, we identified non-O donor-recipient group as an independent risk factor for TEE development after cGVHD compared to the O-O ABO group. This association has not been previously reported and warrants further investigation of ABO blood group effect on risk of thrombosis after allogeneic HCT, specifically in high-risk patients.

Our study identified a subgroup of allogeneic HCT recipients at a high risk for TEE. If this subgroup of allogeneic HCT recipient could be identified prior to the development of TEE, they could be treated with early thromboprophylaxis and other supportive care strategies for prevention of TEE. Biomarkers of endothelial dysfunction after cGVHD could identify the subgroup of allogeneic HCT recipients at risk of developing TEE that would benefit most from intervention.

## Supplementary information

AJ checklist
